# The Interaction between Lockdown-Specific Conditions and Family-Specific Variables Explains the Presence of Child Insomnia during COVID-19: A Key Response to the Current Debate

**DOI:** 10.3390/ijerph182312503

**Published:** 2021-11-27

**Authors:** Royce Anders, Florian Lecuelle, Clément Perrin, Swann Ruyter, Patricia Franco, Stéphanie Huguelet, Benjamin Putois

**Affiliations:** 1 EMC Laboratory, University of Lyon 2, 69500 Bron, France; 1.adresse.prof@gmail.com (C.P.); ruyter.swann@outlook.fr (S.R.); 2 Institut de Psychologie, University of Lyon 2, 69500 Bron, France; 3 Lyon Neuroscience Research Center, CNRS UMR 5292-INSERM U1028, University of Lyon 1, 69000 Lyon, France; florian.lecuelle@unidistance.ch (F.L.); patricia.franco@chu-lyon.fr (P.F.); benjamin.putois@unidistance.ch (B.P.); 4 Pediatric Clinical Epileptology, Sleep Disorders and Functional Neurology, Hospital for Women Mothers and Children, CHU of Lyon, 69500 Bron, France; 5 Faculty of Psychology, Swiss Distance Learning University, 1400 Brig, Switzerland; stephanie.huguelet@unidistance.ch

**Keywords:** sleep disturbance, young children, COVID-19, SARS-CoV-2, lockdown, health crisis

## Abstract

It is still debated whether lockdown conditions in response to the coronavirus disease 2019 (COVID-19) health crisis seriously affected children’s sleep. For young children, some studies identified more insomnia, while others only transient disturbances, or even no effect. Based on the premise of mother–child synchrony, a well-known dynamic established in child development research, we hypothesized that principally, the children whose mothers perceived the lockdown as stressful and/or responded maladaptively, suffered sleep disturbances. The main objective of this study was to identify the family profiles, variables, and lockdown responses most linked to insomnia in young children. The sample consisted of 165 mothers, French vs. Swiss origin (accounting for different lockdown severities), of children 6 months to 5 years old. Validated sleep, stress, and behavior scales were used. Multiple regression, age-matched clustering, and structural equation modeling analyses provided evidence that insomnia in young children is indeed strongly linked to the mother’s reaction to the pandemic and lockdown. Specifically, reactions such as COVID-19 fear/anxiety and obsessive COVID-19 information seeking coincide with heightened vigilance, cascading into reduced child social contact, outings, and increased screen viewing, ultimately culminating in child insomnia and behavioral problems. Mother education level and child day care quality (e.g., home-schooling) were also identified as strong insomnia predictors.

## 1. Introduction

Do health crisis response measures, such as the lockdown conditions of the coronavirus disease 2019 (COVID-19) pandemic, significantly lead to sleep disturbances in young children? Current evidence is controversial. The preliminary studies that took place in Italy identified a weak impact [1], and then in following participants during four weeks, suggested they are transient [2]. Subsequent studies that took place in a mix of countries generally corroborate the observation of a delayed sleep phase, but are conflictual with regard to a negative sleep impact [1,3,4,5,6]. For example, Liu et al. [5] in China found a reduction in sleep disorders in 4–6 year-olds, and Cerasuolo et al. [6] in Italy found an improvement in overall sleep quality also in this age group, but not for children 0–3 years old (in whom sleep was stable). In contrast, Lecuelle et al. [7] in France found a 22% increase in sleep disorders in the young child age group, and Bruni et al. [8] in Italy also found disturbances in young children for sleep variables (e.g., in duration, falling asleep, night awakenings, and parasomnias). What factors or explanations may account for these differing results? The present work aims to provide said response through an interaction-dependent hypothesis, while also addressing how lockdown conditions may principally disturb children’s sleep, specifically in regard to the young child age group.

According to most recent research, the COVID-19 pandemic and associated lockdown measures have had a very significant impact on mental health on a worldwide scale [9,10,11,12]. Sleep difficulties in adults have also been linked to the health crisis [13,14], which can in turn worsen mental health or be linked bidirectionally [15]. Women have been found to be impacted more than men [16,17], specifically with regard to depressive symptoms, stress, anxiety, and degrees of post-traumatic stress disorder [18,19]. In this respect, especially for young children, the mother–child bond might explain how mental health issues and/or sleep disorders are transferred. Indeed, Markovic et al. [20] identified a relationship between the degree of sleep disturbance in young children and stress of their primary caregiver during lockdown. Our interaction-dependent hypothesis includes that mothers may interact differently with lockdown conditions based on their resiliency, coping strategies, and risk factors demographically and culturally, as well as the varying degree of severity of different countries’ lockdown conditions [21,22] and/or fear- invoking media coverage [23,24,25]. Following from this hypothesis, we conjecture that not sufficiently taking into account enough of these variables may confound analyses, and hence explain the conflicting results in the COVID-19 literature.

Parent–infant synchrony is defined as a child’s natural tendency to synchronize with their social and familial environment [26]. This synchronization has been observed, notably between mother and child, on both behavioral (e.g., mirroring of facial expressions, gestures [26]) and physiological levels (e.g., cardiovascular activity [27,28], adrenocortical functioning, and skin conductance [29]). Appropriate parent–infant synchrony is known to facilitate the development of a child’s emotional self-regulation capacity [30]. As a consequence, excessive parental stress is known to impede the healthy development of this intuitive auto-regulation, or “self-soothing” capacity, in infants [31]. Excessive stress from the health crisis, for example, could antagonize the healthy development of self-soothing in young children, in turn increasing the risk of behavioral insomnia.

The transactional model of sleep proposed by Sadeh and Anders notably describes this relationship between sleep development in young children and these complex bidirectional parent–child interactions (e.g., distal and proximal factors) [32]. Therein, sleep regulation in young children is qualified as being mediated by the parent–child relationship and is influenced by the intrinsic and extrinsic context of the child. In this regard, parenting behaviors and reflexes toward their child may influence the child’s quality of sleep, in which these behaviors themselves are also mediated by the cognitive and emotional states or experiences of the parents [33]. For example, in the case of maternal depression, the mother may be more likely to develop a hypervigilance toward her child’s nocturnal awakenings, in turn excessively reacting and increasing the number of parent–child interactions and doing so with notably additional dynamics. This specific example is indeed a type of behavior that has been identified in previous research to induce the development and maintenance of sleep disorders in young children [34]. Indeed, Di Giorgio et al. [1] in Italy linked the COVID-19 pandemic to increased emotional and self-regulation difficulties in both mothers and children. Therefore, one could conjecture that mainly the children of mothers who perceived the lockdown as stressful would suffer sleep disturbances. However, the literature remains controversial on this point as well: for example, a study in Israel [35] found that although the lockdown exacerbated stress and sleep disturbance in mothers, the majority of children in the study did not exhibit sleep disturbances. What could explain the conflicting results in these studies, and how parent–infant synchrony operated differently within them?

Firstly, not all families interact with quarantine and lockdown conditions similarly (e.g., socioeconomic variables, education), nor are they always subjected to the same quarantine severity/restrictions (e.g., countryside vs. metropolitan area, and crucially differ by country). Therefore, families even within the same study may not be equally comparable when these influential variables are not explicitly taken into account, which could create noise in the analyses or lead to Type I and II statistical errors. For example, Suveg et al. [36] in the U.S. demonstrated that the extent of mother–child synchronization and child emotional self-regulation can depend on family adversity and risk variables (e.g., family context, socioeconomic status, parental psychopathology, and so forth), which could explain the conflictual results mentioned in the previous paragraph [1,35]. Therefore, we adopted an interaction-dependent hypothesis for predicting sleep disturbance in young children, notably between family-specific variables (e.g., income, housing, education level, work constraints), leading to different risk profiles as previously noted in [31], and the specific lockdown conditions that, although generally induce anxiety and fear [20,37,38], crucially vary by country, leading to different psychological impacts [9,12].

An important facet of this interaction-dependent hypothesis relies on the ways individual families will respond and (mal)adapt differently to the pandemic and quarantine restraints (e.g., child outings and care quality, parenting styles, remote working). For example, it has been found that the children of parents who work remotely generally have more screen exposure time [39,40], compared to their counterparts who were in daycare nurseries, or watched by another primary caregiver during working hours (and may also have more outings and walks). Given that screens have a deleterious impact on children’s sleep [41,42] and behavior [43,44], this ties into our interaction-dependent hypothesis, where certain families may be more at risk/probable to respond maladaptively to the health crisis, at least with respect to their children. As screen exposure is strongly linked to sleep disturbance, it is arguably a crucial explanatory variable to account for, since moreover, a large number of COVID-19 studies observed increases in screen exposure for all age groups during lockdown [5,7,8,40]. As screen exposure was not controlled for in a number of COVID-19 studies [2,6,20,45], this variable ties into our response that may explain, at least partially, the conflicting results in the literature.

We previously discussed the healthy development of emotional auto-regulation in children which could be impeded by excessive parental stress (e.g., through the pandemic), but it is equally important to qualify the role that individual-level factors, i.e., depression and anxiety in the mother, could play in child insomnia, and vice versa [35,46]. From a biological perspective, it has been found that an elevated production of cortisol in the mother, a stress hormone that induces a state of hyperarousal, leads to hypervigilance and an increased sensitivity to her child’s night awakenings [47,48,49,50]. From a psychological perspective, such hypervigilance might be a product of the mother’s parenting education [51] and/or the confidence in her ability to adequately meet the needs of her child [34,52]. As this dynamic can set in a perpetual negative cycle, the directionality of these relationships is still debated in the current literature [53]. Nonetheless, it is well-accepted that a child’s sleep disorder is a source of parental stress [54,55,56], whether or not initially caused by their depressive symptoms, and this has been corroborated in a more recent study, where it was found that mothers of children who have difficulty sleeping were significantly more stressed than their counterparts [57].

While the absence of integrating various family- and lockdown-specific variables in analyses may account for contrasting results in the COVID-19 literature with regard to sleep disturbance, differences in methodological approaches and measurement also play a significant role. For instance, not all studies specifically evaluated insomnia, yet this is arguably the most crucial sleep variable to carefully evaluate for several reasons. Insomnia is well-known to be sensitive to the effects of psychological contexts [58,59], and moreover, it is more readily compatible to consider it being modulated by lockdown conditions as opposed to more genetically related issues such as respiratory disorders or hyperhidrosis (excessive sweating). The Sleep Disturbance Scale for Children (SDSC) [60] comprehensively evaluates insomnia, and so far, all COVID-19 studies that utilized it found a negative lockdown impact in young children [1,7,8]. In contrast, the Children’s Sleep Habits Questionnaire (CSHQ) [61] also includes behavioral and contextual questions related to sleep, such as “afraid to fall asleep in the dark, afraid to fall asleep alone, falls asleep with rhythmic movements, needs special object”, which may be quite unrelated to insomnia, and indeed, results are mixed for studies that employed this scale [5,6,20,61], in which absence or even a positive lockdown effect was found in some cases [5,6]. Finally, some studies focused more on changes in children’s circadian rhythms or sleep phases [6,35], rather than a full report on insomnia severity such as with the SDSC.

The main objective of the present study was to provide a key response that can explain the controversial literature on whether the COVID-19 health crisis and lockdown conditions negatively impact sleep quality in young children, herein with regard to insomnia specifically. We aimed to achieve this by simultaneously accounting for (through a large transversal study, and advanced data modeling analyses) a maximum of the key potential variables that could be confounding when not taken into account, as determined by a comprehensive literature review on the related topics. Notably, the four-part breakdown of the objectives of this study was to (i) determine whether the severity of lockdown conditions (e.g., herein France vs. Switzerland) can partially explain such disturbances. In this regard, one would expect greater degrees of child insomnia in France. Secondly, (ii) the aim to identify the main factors related to child sleep disturbance during a pandemic response. For example, variables such as child screen time, number of outings, number of family members at home, financial impact, and so forth may play a significant role. Thirdly, (iii) the aim to differentiate the profile of mother–child dyads who have been impacted by the confining lockdown conditions, from those who have not been affected. Based on parent–infant synchrony previously discussed, one may expect the children of mothers who have high scores of anxiety, insomnia, a negative lockdown experience, excessive media information-seeking, and a COVID-19 infection risk to have higher levels of insomnia and behavioral problems themselves. Fourthly, (iv) the objective to understand the inter-relationship of family–mother–child factors that may be strongly linked to insomnia levels in young children, in which all subobjectives culminate in the identification of the most crucial protective and risk factors associated with child sleep quality in the event of a health crisis. Through path analysis modeling, we aimed to quantify the interplay between variables in an integrative model; for example, one may hypothesize a negative financial impact (or job loss) brought on by the pandemic to lead to anxiety in the mother, in turn exacerbating her degree of insomnia and this cascading into greater child insomnia. Note that herein, sleep disturbance is quantified through the previously discussed SDSC, specifically via the insomnia subscale, known as DIMS: Disorders of Initiating and Maintaining Sleep.

## 2. Methods

### 2.1. Participants

The data were collected by administering, in online format, a multi-questionnaire survey to partner nurseries in France and Switzerland. The survey was completed by mothers of children aged between 6 months to 5 years old, and was available between 5 May 2020 to 6 June 2020. Participants first had to read and agree to a detailed consent form before they could begin the survey. The survey was completed anonymously, in which participants chose an alias. Participation in the study was voluntary and unpaid.

The experimental design of including participants living in different countries (here France and Switzerland) provided an opportunity to account for a potential interaction effect based on the differing severity of lockdown conditions between countries. For example, the imperative lockdown in France imposed much more time at home, e.g., leaving home was only permitted for obtaining groceries or for brief, solo sport activities within 5 km of home, contingent on carrying a signed, permission certificate. In contrast, only a partial lockdown was mandated in Switzerland, e.g., individual travel was allowed without need for a justification, contingent on respecting social distancing, and gatherings of up to 5 people were permissible.

### 2.2. Materials and Design

As detailed below, the multi-questionnaire survey included validated scales for measuring sleep disturbance, anxiety, and behavior issues in mothers and/or their young children, and non-standardized questionnaires to collect data on lockdown variables.

The Insomnia Severity Index (ISI [62]) was used to measures the degree of insomnia in mothers. The ISI consists of 7 items, each on a 5-point Likert scale (from 0 to 4), and thus in total, possible scores range from 0 to 28, where higher scores indicate more severe insomnia. A score of 14 is considered a threshold, and scores above it suggest a pathological level of insomnia. Herein, a Cronbach’s α = 0.84 was observed for the ISI (see [63] for more information on this statistic).

The Sleep Disturbance Scale for Children (SDSC [64]) was used to measure the degree of sleep disturbance of children. The SDSC consists of 22 items, each on a 5-point Likert scale (from 1 to 5), and thus in total, possible scores range from 22 to 110, where higher scores indicate more severe sleep disturbance. These 22 items are divided into 5 subscales of specific sleep disturbance types (disorders of: insomnia, hyperhidrosis, respiration, parasomnia, non-restorative sleep, and excessive somnolence). The SDSC has proven to have strong psychometric validity, with a high internal consistency of 0.79 among control participants and 0.71 among clinical participants [65]. As advocated in the Introduction Section, to account for the most relevant type of sleep disturbance to be potentially impacted by lockdown conditions, we utilized the results specifically of the insomnia subscale of the SDSC, known as Difficulties Initiating and Maintaining Sleep (DIMS). The DIMS subscale is composed of 8 items, such as number of hours the child sleeps (1 = 9–11 h; 5 = less than 5 h), and 7 items that also measure problems initiating sleep and maintaining sleep: time to fall asleep on average (1 = less than 15 min; 5 = more than 60 min), and 6 used a frequency scale (1 = never; 5 = always/daily). These 8 items lead to a possible total score of 8 to 40, where a score higher than 16 suggests a pathological level of insomnia. Herein, a Cronbach’s α = 0.79 was observed for the SDSC, and α = 0.90 for the DIMS subscale.

The Conners’ Global Index (CGI-P [66,67]) was used to measure the degree of behavior issues and hyperactivity in children. The CGI-P consists of 10 items, each on a 4-point Likert scale (from 0 to 3), and thus in total, possible scores range from 0 to 30, where higher scores indicate more behavior issues and hyperactivity. A score of 16 is considered a threshold, and scores above it suggest a pathological level of behavior hyperactivity. Herein, a Cronbach’s α = 0.88 was observed for the CGI-P.

The State Trait Anxiety Inventory form Y-B (STAI-B [68]) was used to measure the degree of anxiety in mothers. The STAI-B consists of 20 items, each on a 4-point Likert scale (from 1 to 4), and thus in total, possible scores range from 20 to 80, where higher scores indicate more anxiety. A score of 60 is considered a threshold, and scores above it suggest a severe level of anxiety. Herein, a Cronbach’s α = 0.78 was observed for the STAI-B.

A COVID-19 pandemic and lockdown response questionnaire was composed and utilized (see Supplementary Table S1) in order to collect important and relevant data pertaining to: the mother’s psychological reaction to the context (e.g., fear, vulnerability, infection proximity, information seeking), family organization and impact (e.g., home-schooling, remote working, financial impact, screen viewing), and demographic variables (e.g., mother’s age, education level, housing, child gender).

### 2.3. Data Pre-Processing

The data were analyzed and pre-processed using the R programming language, with Rstudio as the coding environment (Version 1.1.463). Outliers were identified based on a linear model-based approach using Cook’s distance (values > 0.04 [69]). In order to satisfy modeling and statistical test criteria (e.g., normality), the noncategorical variables were optimally normalized via the Yeo-Johnson transformation [70]. Preliminary analyses (Student’s *t* test for continuous/interval variables, Wilcoxon rank sum test for ordinal variables, and χ^2^ frequency test for binary variables, as well as principal component analyses) were used to reveal significant differences between the French and Swiss participants, ascertaining that the differing lockdown conditions of the two countries (a confound) leads to their respective different interactions along the variables. As a result, henceforth the sample is evaluated along these two groups in order to provide a more fine-grained analysis. Finally, as the data involved an excessive number of potential explicative variables (>30), a standard backward stepwise regression analysis was used to identify the most relevant variables for further analysis.

### 2.4. Data Analysis

Standard descriptive statistics were calculated on the variables in which *p*-values less than 0.05 were considered to be statistically significant. In line with a number of comparable COVID-19 studies [1,3,8] discussed in the Introduction Section, that had not corrected for multiple comparisons on the basis of the exploratory nature of their study, the present study (involving a comparable sample size, and a multitude of variables) followed similarly. First, correlational and principal component analyses were carried out to identify highly impactful vs. redundant variables, and how they may group. Second, backward stepwise linear multiple regression models were realized for each country (*stats* package in R, version 4.1.1, R Foundation for Statistical Computing, Vienna, Austria). Third, a high-performance hierarchical clustering of the participants was obtained by directly using the principal component information (*HCPC* package in R). Finally, path analysis was performed within the structural equation modeling (SEM) framework in order to obtain an integrative network model that details the relationships between the variables for each country (*lavaan* package in R). No latent explicative/composite variables were added into the model. The resultant networks were obtained through a data-driven approach: optimizing a Bayesian network structure learning algorithm (*bnsl* package in R). In each case, the simplest SEM network structure that appropriately satisfied the standard reference SEM diagnostics (e.g., Tucker-Lewis Index (TLI), Bollen’s Incremental Fit Index (IFI), Root Mean Square Error of Approximation (RMSEA)); see Table S2 in Supplementary Materials) was selected. Then, the plausibility of several additional paths was evaluated (e.g., theoretically motivated, or suggested by the standard modification indices provided through the *lavaan* package), and these were retained only if they substantially improved the SEM diagnostics.

## 3. Results

### 3.1. Participants

The analyses herein consisted of 165 participants, after a filtering from an original 210 participants, based on full completion of the questionnaire (N = 18) and outlier detection and elimination (N = 27) detailed in the Methods Section. These 165 participants consisted of 81 French and 84 Swiss mothers, between 26 and 52 years old (mean = 36.06; SD = 4.26), of children between 7 to 70 months (mean = 34.2; SD = 15.2). The average (SD) age, respectively, of mothers and children for the French sample was 35.14 (3.81) years old and 32.54 (14.62) months old (36% male), and for the Swiss sample, 36.63 (4.62) years old and 34.13 (15.73) months old (56% male). More detailed descriptive statistics comparing the samples are reported in Table S3 (Supplementary Materials).

### 3.2. Linear Multiple Regression on Child Sleep Disorder and Insomnia (DIMS)

For each country, a backward stepwise multiple linear regression modeling was performed to quantify the potential predictive relationships of each collected variable with regard to the degree of child insomnia (DIMS). In this approach, initially, all explicative variables are included in the model; then, iteratively, the least predictive variables for DIMS are removed one-by-one from the regression model until the removal of the next least-predictive variable would worsen the goodness of model fit (here quantified by the Akaike Information Criterion, AIC [71]).

#### 3.2.1. French Sample 

The results of the modeling for the French sample are included in Table 1, for which a significant regression equation was found with an *F*(18,72) = 15.97 and *p* < 0.001 (*R²* = 0.82, adj. *R²* = 0.77). For the French sample, child sleep disorder impact on the family, child behavioral problems (CGI-P), female gender (coded as 1), proximity to an infected person, lockdown impact (coded –1 = positive, 0 = neutral, 1 = negative), and older mothers were all significantly associated with more child insomnia, while in contrast, more cohabitants in the household, seeing friends more, and the mother knowing someone infected with COVID-19 were significantly associated with less child insomnia.

#### 3.2.2. Swiss Sample

The results of the modeling for the Swiss sample are included in Table 2, for which a significant regression equation was found with an *F*(10,73) = 18.11 and *p* < 0.001 (*R²* = 0.71, adj. *R²* = 0.67). For the Swiss sample, child sleep disorder impact on the family, financial impact, mother insomnia, higher education level, and COVID-19 fear were all significantly associated with more child insomnia, while in contrast, mothers who worked more and had higher risk of infection were significantly associated with less child insomnia.

### 3.3. Different COVID-19 Response Groups Based on Clustering Analysis

In the Introduction Section, we proposed an interaction-dependent hypothesis that individual mothers/families, based on their psychological, socioeconomic, and demographic variables, may interact differently with the specific lockdown conditions in their country, leading to varying susceptibility to mother/child insomnia. In order to further investigate this conjecture, we performed an advanced hierarchical clustering analysis on the principal component decomposition of the data for each country.

#### 3.3.1. French Sample

**Figure 1:**
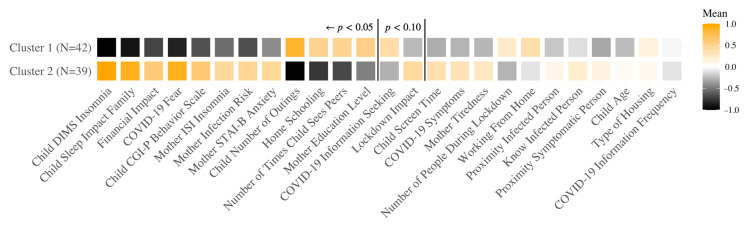
Hierarchical clustering analysis results for the French sample. Each square represents the average value of the corresponding variable (transformed Yeo-Johnson) [70]. Brighter colors indicate high values while blacker colors indicate low values. The variables are ordered based on the most significantly different, positively, between the clusters, then negatively, then the non-significant differences. Variables to the left of the first vertical line are significantly different between the clusters. DIMS: Difficulties Initiating and Maintaining Sleep; CGI-P: Conners’ Global Index; ISI: Insomnia Severity Index; STAI-B: State Trait Anxiety Inventory form Y-B.

The optimal result of the hierarchical clustering algorithm led to two groups in the French sample (Figure 1). For each cluster, we calculated the mean values for each variable (after a Yeo-Johnson, standardized transformation, to ensure comparable scales for visualization). Particularly, the analyses resulted in a symptomatic or risk cluster, and an asymptomatic or well-adapted cluster. Specifically, the two clusters crucially differ (in ranked significance order, Cluster 2 positive differences) on the degree of child insomnia (DIMS), child sleep disorder impact on the family, pandemic financial impact, COVID-19 fear, child behavioral problems (CGI-P), mother insomnia (ISI), mother infection risk, and mother anxiety (STAI-B), and (Cluster 2 negative differences) number of child outings, home-schooling, child seeing peers, and mother education level. Table S4 in the Supplementary Materials provides the respective means (SD) for each cluster and the two-sample *t*-test significance levels between clusters. The two clusters are not significantly different in child age (*t*(38) = −0.65, *p* = 0.52) or gender (χ^2^(1) = 2.80, *p* = 0.09), and thus these variables did not play a significant role in determining the clusters, or their differential degree of pathology (e.g., child insomnia).

#### 3.3.2. Swiss Sample

**Figure 2:**
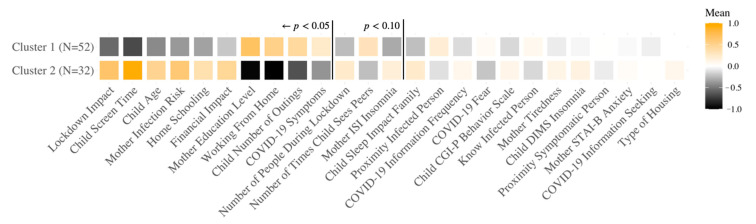
Hierarchical clustering analysis results for the Swiss sample. Each square represents the average value of the corresponding variable (transformed Yeo-Johnson) [70]. Brighter colors indicate high values while blacker colors indicate low values. The variables are ordered based on the most significantly different, positively, between the clusters, then negatively, then the non-significant differences. Variables to the left of the first vertical line are significantly different between the clusters. ISI: Insomnia Severity Index; CGI-P: Conners’ Global Index; DIMS: Difficulties Initiating and Maintaining Sleep; STAI-B: State Trait Anxiety Inventory form Y-B.

The optimal result of the hierarchical clustering algorithm led to two groups in the Swiss sample, for which the results are visualized in Figure 2. Likewise, the analysis led to a more symptomatic or risk cluster vs. a better-adapted cluster. Though importantly, while the Swiss symptomatic cluster expressed being impacted by the lockdown, in contrast to the French sample clustering analysis, child insomnia was not a significant factor differentiating these clusters. Rather, the clusters crucially differ (in ranked significance order, Cluster 2 positive differences) on the basis of lockdown impact, child screen time, child age, mother infection risk, home-schooling, and financial impact, and (Cluster 2 negative differences) mother education level, working from home, number of child outings, and COVID-19 symptoms. Table S5 in the Supplementary Materials provides the respective means (SD) for each cluster and the two-sample *t*-test significance levels between clusters. Unlike the French sample, here, the two clusters are significantly different in terms of child age (*t*(38) = −3.20, *p* ≤ 0.01), though still not gender (χ^2^(1) = 4.11 × 10^−31^, *p* = 1), and thus age may have played a significant role in determining these clusters, with a magnitude on par with the impact of the Swiss partial lockdown conditions.

In summary, the population samples from both countries indeed demonstrate an impact of the COVID-19 pandemic and lockdown, yet differently; crucially, child sleep quality is more disturbed in the French sample than in the Swiss sample (*t*(80) = −5.71, *p* ≤ 0.01). For both countries, of the two clusters, one cluster exhibited a negative impact of the pandemic and the variables associated with it, while the other demonstrated more effective conditions or coping strategies to manage the impact of the country’s specific lockdown situation.

### 3.4. Integrative Model of COVID-19 Variable Relationships through Structural Equation Modeling

In this section, SEM analyses were performed in order to reveal the relationships between the key variables identified in the clustering analyses, simultaneously, in the context of a single integrative model. The principal network structure of the model was obtained through a data-driven approach, specifically by the convergence of a Bayesian network structure machine learning algorithm.

Consistent with our interaction-dependent hypothesis, the clustering analyses demonstrated group-level consistent trends of mothers/family types that interact, or were able to respond adaptively to the pandemic, hence reducing insomnia risk, vs. those that responded maladaptively, and what those key protective and risk variables consist of. So far, the analyses have shown that the French sample, having undergone full lockdown measures, were vulnerable to mother/child insomnia, whereas in the Swiss sample having undergone a partial lockdown, rather variables less central to the pandemic itself differentiated the clusters, such as child age, mother education level, and child screen time. For these reasons, in this section of the SEM analysis, we focus only on the French sample, also because the SEM for the Swiss sample did not provide new elements for discussion over the initial regression and clustering analyses (however, the Swiss sample SEM is provided in Figure S1 of the Supplementary Materials).

#### French Sample

As provided in Table 3, the resultant French SEM satisfied the majority of the standard fit diagnostics, specifically 8 out of 10 thresholds. Particularly, the Goodness of Fit Index (GFI) and the Adjusted Goodness of Fit Index (AGFI) were not satisfied, however these statistics have been discouraged on the basis that they are known to be strongly affected by sample size (see [72]). As complementary fit information, the observed R² values for the key variables were the following: Child DIMS Insomnia (0.33), Mother ISI Insomnia (0.32), Child Sleep Impact Family (0.53), Mother STAI-B Anxiety (0.38), Mother Tiredness (0.52), Child CGI-P Behavior Scale (0.26), and COVID-19 Fear (0.22).

**Figure 3:**
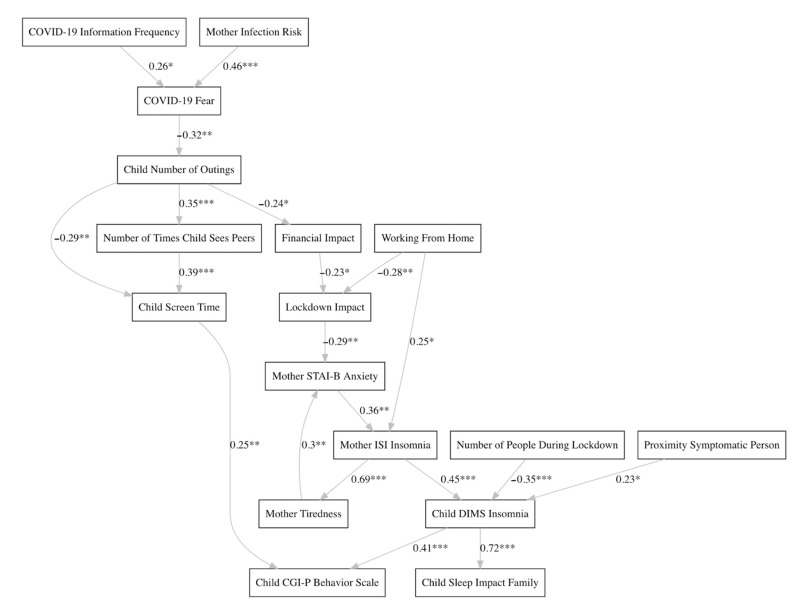
Path analysis for the French sample using the structural equation modeling framework. The principal network structure was derived through a data-driven, Bayesian network structure learning approach. Significance levels of the relationships modeled: *** *p* < 0.001, ** *p* < 0.01, and * *p* < 0.05.

The results of the SEM analysis suggest that COVID-19 information seeking and mother infection risk contribute to the mother’s fear of COVID-19 (Figure 3). In turn, more COVID-19 fear may influence the mother to allow fewer child outings. Naturally, child outings may increase the number of times children see their peers, and decrease screen time. However, when children do see their peers, notably in a lockdown context where few recreational activities are available, the SEM results suggest that this may rather lead to more child screen time (watching movies together, video games). More child screen time is linked to child behavioral problems/hyperactivity, which is also importantly linked to child insomnia. The SEM results also indicate that families who take out their children tend to be those who have been less impacted financially by the COVID-19 pandemic.

The valence of lockdown impact appears to be importantly related to what extent a family is financially affected by the pandemic, and if the mother can still work from home (hence make a living). A negative lockdown impact (coded as 1) is linked to the mother’s anxiety level, which in turn is tied to her insomnia level. The mother’s insomnia may naturally result in more tiredness, and a vicious cycle is suggested by the model: whereas she becomes more tired, this leads to more anxiety, again exacerbating her degree of insomnia. Mothers who work from home tend to have more insomnia, which may correspond to less work–life balance.

Crucially, the SEM results support the hypothesis that the child’s insomnia is closely linked to the mother’s insomnia. The mother being in proximity to a symptomatic person also showed a relationship to more child insomnia, suggesting a transfer of the mother’s uneasiness to the child (related to parent–infant synchrony discussed in the Introduction Section). More people in the household during lockdown was linked to less child insomnia, inviting an interpretation of shared care/rearing responsibilities and more social interaction for the child. Finally, child insomnia is strongly linked to the impact of the child’s sleep problems on the family’s quality of life.

## 4. Discussion

### 4.1. Identifying a Potential Source of the Controversial Findings

A principal objective of the present study was to offer an explanation for the origin of the controversial findings in the current literature about the presence [1,2,7] vs. absence [5,6] of lockdown-induced sleep disorders in preschool (4–6 years) children, or even improvements found in toddlers (<3 years) [6]. We aimed to provide said response through taking into account the important, as well as the potential confounding, variables with respect to lockdown conditions (e.g., lockdown severity, via French vs. Swiss population comparisons), family variables (socioeconomic, size, remote working, housing, context), and the mother’s psychosocial dynamics (fear, anxiety, insomnia, tiredness).

### 4.2. Interaction between Lockdown and Family Variables Predicts Insomnia in Young Children

Our results provide one of multiple explanations for the origin of the controversial findings. Firstly, it was found that the lockdown conditions of the French and Swiss samples were different enough that the predictor variables of insomnia interact differently. The difference in sleep disturbance outcomes that can occur based on differing countries’ lockdown severities has already been shown in two previous studies in adults (France and Greece) [21,22]. The statistical consequence is that if two such countries’ population samples are grouped together in the same statistical tests, many of the significant variables would be drowned out by the noise of their interaction, which would prevent knowing the real impact of each lockdown [20]. The same could be argued based on clusters of individuals, who might interact differently with the pandemic/lockdown conditions.

In this regard, our explanation for the debate is further corroborated by our clustering analyses, which identified two subpopulations respectively within each country that responded differently to the lockdown conditions, resulting in a more symptomatic or risky group and a less symptomatic group (i.e., that may employ more effective coping strategies). Hence, if another study obtains a pathological group (e.g., a DIMS score greater than the pathological threshold [64,82]) that is too small (e.g., by population sampling variability or sociocultural factors), and the data also include many individuals that employ effective coping strategies for example, the probability to successfully detect a significant effect in certain risk/causal variables as related to the COVID-19 pandemic may be reduced.

In each country, the pathological group is explained by weaker valence in the protective variables (e.g., child outings, social contact) and higher valence in the risk variables (e.g., financial impact and other COVID-19 variables), and a number of these variables are corroborated in previous research [8,20]. The strongest protective and risk variables tend to differ by country, as well as the presence of lockdown-induced sleep disorder in young children. For example, the more pathological cluster in France consists of significant degrees of child insomnia (DIMS), child behavioral problems (CGI-P), mother insomnia (ISI), mother anxiety (STAI-B), and mother COVID-19 fear, where the child seeing peers and having more cohabitants are uniquely significant protective factors compared to the Swiss pathological cluster. In contrast, the Swiss pathological cluster did not exhibit significant degrees of child insomnia nor behavioral problems compared to its counterpart cluster, and mainly child screen time, child age, and overall lockdown impact perception appear to distinguish the clusters (e.g., their degree of insomnia), and is less explained by specific lockdown variables (in comparison to the French). This is coherent with the fact that Swiss residents lived a semi-lockdown (free individual travel, gatherings of less than 5 people allowed), compared to the full lockdown for French residents (leaving home permitted only for groceries or compelling motive).

Therefore, our results corroborate findings in a number of previous studies, and furthermore, support our hypothesis that pandemic-induced insomnia in young children is predicted by an interaction between lockdown- and family-specific variables, in a biopsychosocial framework [8,9,20], that for example, give rise to different ways individuals may respond and (mal)adapt to a health crisis and the associated government-imposed measures.

### 4.3. Explaining the Main Factors Influencing Child Sleep Disturbance during a Pandemic Response

In France, the results of the clustering and multiple regression models demonstrate that sleep disorders in young children are mainly negatively associated with the mother’s COVID-19 fear, her anxiety, her insomnia, child hyperactivity, and the pandemic’s financial impact on the family. In the regression model, the mother knowing someone infected with COVID-19 was related to lower child insomnia, suggesting that such a personal rapport (e.g., where communication by phone, email, and internet is possible) may be informative, leading to a more realistic experience of the dangers associated with the disease rather than being too easily influenced by (e.g., fear-invoking) social media or unfounded personal hypotheses. In contrast, the proximity variable in the model measured specifically the extent to which the mother is habitually in close physical proximity to an infected person, and as expected (as a source of stress), was linked to higher child insomnia. Relationships were also observed between the child’s insomnia and his/her number of outings, home vs. regular schooling, and the mother’s education level. These results are in support of established theories on the bidirectional relationship of the mother’s well-being and the child’s quality of sleep [83]. In this respect, our results invite the interpretation that greater infection risk for the mother may provoke more fear of COVID-19, in turn generating more pandemic-related anxiety and insomnia, where cautious behaviors are produced, such as fewer child outings, therefore cascading into increased child hyperactivity and sleep disturbance. This interpretation is also supported by the SEM results discussed later, and elements of this modeling are corroborated by results of a previous study, in older children (9–12 years) [38]. Furthermore, financial impact was also found to be strongly linked to the mother’s anxiety (see [84]), which is well-known to lead to sleep disorders [85], and financial impact was also found to be significantly related to a negative overall perception or experience of the lockdown (variable: Lockdown Impact).

In Switzerland, the clustering analysis results suggest that the population subgroups differ more on lockdown perception (Lockdown Impact) rather than its impact on child sleep. Specifically, this Lockdown Impact variable measured (mother’s perception) to what degree the lockdown has a positive, neutral, or negative impact (coded in our analyses as higher variable values) on her quality of life. According to our results in the Swiss sample, a positive lockdown perception was associated with lower times of screen use in children, a higher mother education level, and remote working. These three variables were related to lower financial impact and lower infection risk, which in turn are related to reduced mother anxiety. A higher education level in mothers may also lead to more effective coping strategies. In this way, these mothers may be better informed to protect themselves and their children from being contaminated, accord an importance to their children seeing their peers, and control their screen viewing time (well-known to impact children’s sleep [41,42]). Consistent with this result, the regression model also found that children of Swiss mothers with a higher infection risk (e.g., professional environment) had lower insomnia, suggesting that these mothers may be more informed about the actual risks/appropriate protective protocols of the disease. The child insomnia result was also less for mothers who worked more, dually suggesting they are less financially impacted and may have less time to be influenced by/searching for COVID-19 media—both of which may be linked to lower anxiety levels.

Child sleep quality seems to have been better maintained in the Swiss population, perhaps also in part due to greater opportunities for regular social contact for all, due to them only incurring a partial lockdown. Increased social opportunities could have offered the Swiss more protection against anxiety issues, indeed despite higher proportions of COVID-19 symptoms reported in the population. In this way, the Swiss semi-lockdown may be considered “more appreciable” on a psychological level. This invites the hypothesis that supporting mothers’ psychological factors such as anxiety (key influencers: financial loss, social contact, media information) supersedes COVID-19 symptom/risk management as a key strategy to assuring mother and child insomnia in the perspective of future/multiple lockdowns.

Our results demonstrate that the different lockdown conditions originating from different government responses to the COVID-19 pandemic can indeed lead to different consequences in young children, specifically sleep and behavioral disturbances, as well as psychological challenges (fear, anxiety, depression, insomnia) within the family [86].

### 4.4. Further Identifying the Causal Links through Structural Equation Modeling

In order to model the interplay and mediated relationships between the variables collected (e.g., path analysis), a data-driven network analysis approach was used, based on combining the SEM framework and Bayesian network structure learning.

The SEM for the French sample was particularly consistent with the clustering results previously discussed. The SEM results suggest that the degree of child insomnia (DIMS) is principally linked to the mother’s insomnia (ISI), and may be exacerbated when the child has fewer outings and social contact opportunities. Indeed, the SEM demonstrates that young children with more cohabitants (e.g., family members in the household) have less insomnia, providing shared rearing and additional social contact opportunities for the child. Conversely, when a family member is infected, higher levels of insomnia were identified in these young children, suggesting, like sponges, that they are sensitive to the negative emotions and sounds of the sick family member (see [36]). The child’s number of outings are principally inversely related to the mother’s fear of COVID-19, infection risk, and information seeking about the disease. Specifically, being more at risk to contract the virus and/or more frequently seeking information about it is fear producing, translating into fewer outings/social contact opportunities for the child. When young children suffer insomnia, this in turn is linked to behavioral problems (CGI-P), which are also directly related to the amount of the child’s digital screen use.

Furthermore, the SEM results also support that the mother’s anxiety level is strongly associated with her degree of insomnia. Her anxiety can be tied to her quality of life during the lockdown (strongly determined by financial impact) as well as her tiredness (e.g., due to insomnia), in which the former, recursively, could predict her anxiety. Hence, crucially, a vicious cycle is apparent where as she becomes more tired (for example, unable to meet her daily responsibilities appropriately), this may lead to more anxiety, again exacerbating her degree of insomnia. Indeed, previous studies have corroborated a relationship between the mother’s insomnia and her anxiety level [35,38,85]. In addition, herein, the SEM showed that mothers who work from home tend to have more insomnia, which may suggest difficulty in achieving a healthy work–life balance.

This strong role that anxiety plays (mediated by the mother’s insomnia) culminating into more child insomnia is consistent with parent–infant synchrony research [26]. The SEM herein shows that the mother’s fear of being infected with COVID-19 and COVID-19 information seeking/fear-inducing media [23,24,25] can cascade into anxiety [84], which can in turn lead to insomnia [85]. In agreement with the study by Brooks and collaborators (2020) [9], our results demonstrate that many different lockdown factors may impact the psychology of the mother. Reduced social contact opportunities for the child is an unfortunate reaction related to the mother’s fear, anxiety, or financial impact of the pandemic, resulting in more screen viewing time of the child and ultimately behavioral and sleep issues. The mother’s anxiety, as well as the child’s habituation to screen usage, may hinder his/her natural development to manage their social environment and self-calm. Especially, mothers more strongly impacted by the lockdown risk accentuating their children’s sleep disorders, by transmission of their own lack of emotional well-being, or by radically changing their children’s social and interactional environments.

### 4.5. Strengths and Limitations

The psychometric validity of the questionnaires employed (SDSC, ISI, CGI-P) in the study is well-supported. The SDSC [60,64] measures five specific categories of sleep disturbance (e.g., insomnia, drowsiness, apnea, parasomnia, nonrestorative sleep) and may be preferred over the CSHQ, which measures general criteria. Indeed, the SDSC has been shown to have good internal consistency, and has been validated in multiple languages [87,88,89,90]. Consistent with these findings, herein, a Cronbach’s α of 0.90 was obtained for the DIMS subscale of the SDSC that measured insomnia severity for the current study. Among the 144 questionnaires noted in 2011 [91] for measuring children’s sleep quality, the SDSC (herein appropriately the French-translated version [82]) is the only questionnaire to meet the 11 key sleep criteria proposed by Spruyt and Gozal [91]. These 11 criteria are used to guide the appropriate validation and creation of questionnaires for evaluating children’s sleep quality. In comparison, the CSHQ, although composed of relevant sleep-quality questions, does not include the complete criteria to fully evaluate child insomnia; moreover, not all questions are on the same Likert range (3 vs. 4 points), which can make the interpretation of results more difficult. Indeed, as mentioned in the Introduction Section, mainly the studies that did not identify a negative lockdown impact on child sleep were ones that used the CSHQ [5,6].

Another strength of the present study involves its advanced use of modeling and data analysis approaches (e.g., principal component analysis, multiple regression, hierarchical clustering, and structural equation modeling, as well as appropriate data preparation to satisfy test/model requirements). This allowed for the ensemble of potential predictive variables to be taken into account together (or partitioned accordingly, e.g., by country), and treated appropriately. Together, these analyses provided a potential major explanation for the divergent results that have fueled the current debate on lockdown-induced sleep disturbance in young children. Lastly, the data-driven structural equation model (SEM) analysis presented a powerful integrative model that provided insights into the different relationships and interplays between the variables.

However, the small sample size is a binding limitation that did not allow us to explore more complex versions of the SEM, such as to truly ascertain to what extent the links are bidirectional, to model the interactions, and also, to obtain stronger fit indices. Furthermore, it is also possible that more variables may have been identified in the SEM, or even in the regression or clustering analyses, if we possessed a larger sample. On this note, a number of other studies also involved comparable sample sizes [1,3,6], and this is an important point of improvement for future research on this topic, should the need for full lockdown measures arise again, and also, in order to achieve a more robust SEM analysis. Nonetheless, the sleep impact results of our study corroborate well with those of Bruni and colleagues [5], that showed a strong negative lockdown impact of insomnia in young children (aged 1 to 5 years), with a large sample of over 2000 participants.

As the current study was not longitudinal in the sense that we did not have measures of these variables for the same participants prior to the lockdown context, the study design does not allow one to confirm that the relationships identified are necessarily causal. On this note, it is worthwhile to mention that a previous French study [7] that had analyzed pre–post-lockdown groups (albeit different samples) found a 22% increase in child insomnia (quantified by DIMS scores).

## 5. Conclusions

One in two children may suffer from sleep disorders as a result of COVID-19 lockdown conditions. Although overall there is no primary direct effect of lockdown-induced insomnia in young children, the presence of the effect may be determined by an interaction between the lockdown variables (e.g., country-specific measures) and family variables, for which our study has identified specific relationships. In future work, these variables could be used to effectively predict the probability of a family or mother to employ maladaptive vs. effective coping strategies. Our analyses demonstrate that population subgroups (of young children/mothers) can be identified as most at risk for developing insomnia disorders, and importantly targeted for assistance or clinical intervention. Promising interventions might not only focus on treatment for insomnia but also focus on mitigating anxiety, providing appropriate education to caregivers and on how to deal with COVID-19 media news, more child outings and social interaction, as well as limiting their screen time, appropriate daycare, and financial assistance. To allow for improved cross-cultural meta-analyses, future research would benefit from agreeing on a common measurement methodology, such as employing the SDSC [60], which we advise based on its established interpretative validity. Herein, advanced analytical and machine learning approaches allowed for the extraction of richly informative results, despite working on a modest sample size.

## Figures and Tables

**Table 1:** Linear multiple regression results for the prediction of child insomnia (DIMS subscale of the SDSC, French sample).

Variable	*β*	CI	*p*
Child Sleep Impact Family	0.59	0.46–0.73	**<0.001**
Child Behavior: Conners’ Global Index	0.33	0.20–0.46	**<0.001**
Child Gender	0.23	0.11–0.34	**<0.001**
Proximity Infected Person	0.23	0.10–0.36	**0.001**
Lockdown Impact	0.20	0.07–0.33	**0.003**
Mother Age	0.19	0.06–0.32	**0.004**
Number of People During Lockdown	−0.35	−0.48–−0.22	**<0.001**
Number of Times Child Sees Peers	−0.29	−0.41–−0.16	**<0.001**
Know Infected Person	−0.23	−0.36–−0.10	**0.001**
COVID-19 Information Seeking	0.11	−0.02–0.23	0.09
Working From Home	0.11	−0.02–0.24	0.09
Mother Infection Risk	−0.11	−0.23–0.02	0.09
Mother Insomnia Severity Index	0.11	−0.02–0.24	0.09
Proximity Symptomatic Person	0.10	−0.03–0.23	0.14
COVID-19 Information Frequency	−0.10	−0.24–0.04	0.17
Financial Impact	0.09	−0.04–0.22	0.16
Education Level	−0.08	−0.20–0.04	0.18

Note: Variables before the first separating line are associated with more insomnia, after with less insomnia, and variables after the second line (mixed) are nonsignificant based on *p*-values > 0.05. In the interest of brevity, variables with *p*-values > 0.2 are not included in the table (including the intercept, *β =* −0.01, *p* = 0.80). Significant *p*-values are indicated in bold.

**Table 2:** Linear multiple regression results for the prediction of child insomnia (DIMS subscale of the SDSC, Swiss sample).

Variable	*β*	CI	*p*
Child Sleep Impact Family	0.61	0.47–0.74	**<0.001**
Financial Impact	0.29	0.17–0.41	**<0.001**
Mother Insomnia Severity Index	0.24	0.11–0.37	**<0.001**
Education Level	0.19	0.06–0.33	**0.01**
COVID-19 Fear	0.14	0.02–0.26	**0.03**
Work Duration	−0.15	−0.27–−0.03	**0.02**
Mother Infection Risk	−0.13	−0.26–−0.01	**0.03**
Proximity Infected Person	0.11	−0.00–0.23	0.06
Mother Age	0.12	−0.01–0.24	0.07
Working From Home	−0.10	−0.23–0.03	0.12

Note: Variables before the first separating line are associated with more insomnia, after with less insomnia, and variables after the second line (mixed) are nonsignificant based on *p*-values > 0.05. In the interest of brevity, variables with *p*-values > 0.2 are not included in the table (including the intercept, *β =* 0.05, *p* = 0.40). Significant *p*-values are indicated in bold.

**Table 3:** Observed fit indices and appropriate thresholds for the structural equation model of the French sample.

Indices	Observed Value	Acceptable Threshold
Model χ²/*df* [73]	1.19	<5.0
CFI ^1^ [74]	0.93	>0.90
IFI ^2^ [75,76]	0.93	>0.90
NNFI ^3^ (TLI) [77]	0.92	>0.90
RMSEA ^4^ [78]	0.049	<0.10
RMSEA *p* Close Fit [79]	0.505	>0.10
RMSEA 90% Confidence Interval [79]	[0.00; 0.079]	[0.00; Close to RMSEA]
GFI ^5^ [80]	0.82	>0.90
AGFI ^6^ [80]	0.74	>0.90
Model χ² (*df* = 105) ^7^	125.29, *p* = 0.09	*p* > 0.05
Baseline model χ² (*df =* 126) ^7^	414.47, *p* < 0.001	*p* < 0.05

Note: ^1^ Comparative Fit Index; ^2^ Bollen’s Incremental Fit Index; ^3^ Non Normed Fit Index (also known as Tucker-Lewis Fit Index); ^4^ Root Mean Square Error of Approximation; ^5^ Goodness of Fit Index; ^6^ Adjuted Goodness of Fit Index; ^7^ Minimum function test statistic: null hypothesis corresponds to an ideal model, thus smaller χ² values, and hence *p*-values > 0.05 are preferred for the observed model [73]. See Table S1 in the Supplementary Materials, as well as Schermelleh-Engel and colleagues [81], for more detailed information on the fit indices and their recommended thresholds.

## Data Availability

The data presented in this study are available upon request from the corresponding authors.

## Supplementary Materials

The following are available online at https://www.mdpi.com/article/10.3390/ijerph182312503/s1. Figure S1: Path analysis for the Swiss sample using the structural equation modelling framework. The principal network structure was derived through a data-driven, Bayesian network structure learning approach. Significance levels of the relationships: *** *p* < 0.001, ** *p* < 0.01 and * *p* < 0.05, Table S1: Labels and Measures, Table S2: Description and Preferred Threshold of SEM Fit Indices, Table S3: Descriptive Statistics for All Participants, French, and Swiss, Table S4: Descriptive Statistics for the French Clusters, Table S5: Descriptive Statistics for the Swiss Clusters. References [73,74,75,76,77,78,79,80,81] are cited in the supplementary materials.
