# Supplementary material for: The Interaction between Lockdown-Specific Conditions and Family-Specific Variables Explains the Presence of Child Insomnia during COVID-19: A Key Response to the Current Debate
Source: Int J Environ Res Public Health. 2021 Nov 27;18(23):12503. doi: 10.3390/ijerph182312503 (PMC8656994; doi:10.3390/ijerph182312503)
Supplement: Supplementary file 1 [file ijerph-18-12503-s001.zip › ijerph-1422144-supplementary.pdf]

Supplementary Materials

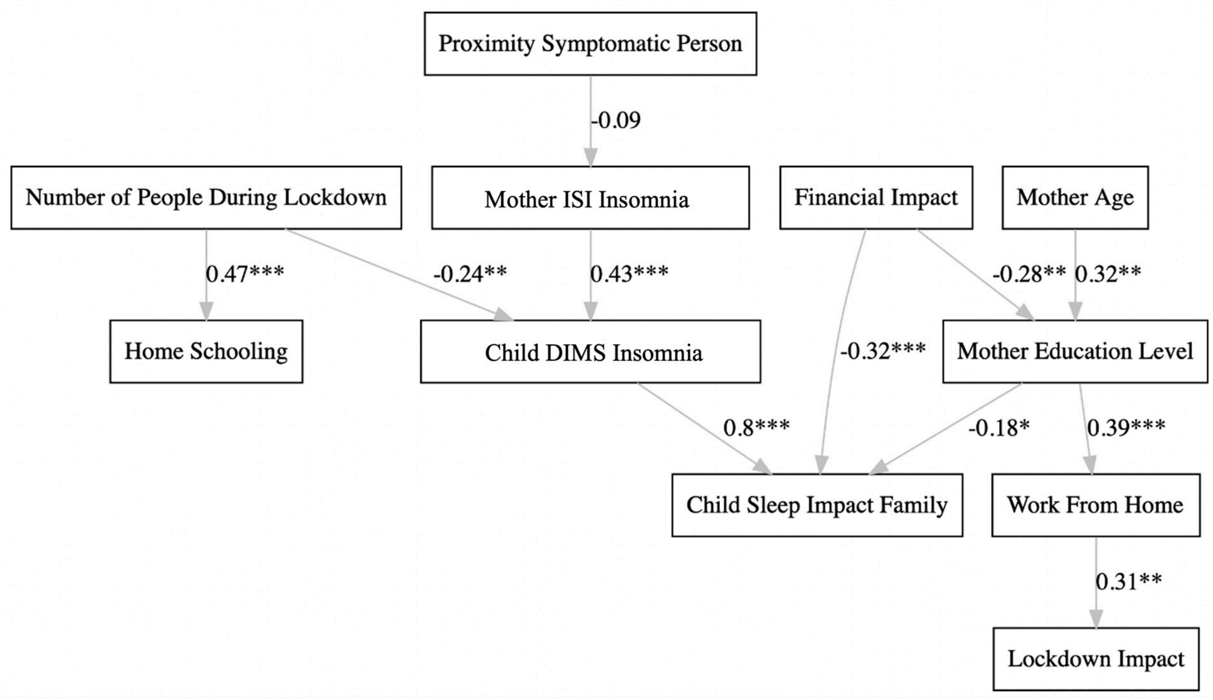

Figure S1. Path analysis for the Swiss sample using the structural equation modeling framework. The principal network structure was derived through a data-driven, Bayesian network structure learning approach. Significance levels of the relationships modelled: *** $p < 0.001$, ** $p < 0.01$ and * $p < 0.05$.

Table S1. Labels and Measures

Variable	Items	Response
Number of People During Lockdown	*The number of people living in the house during lockdown, including yourself?*	Number of adults, number of children
Lockdown Impact [1]	*Do you believe that the lockdown had an impact on you or your child?*	Yes/No + Free Comments
Mother Infection Risk	*Are you regularly at risk to contract the disease based on work or other situations?*	Yes/No
Know Infected Person	*Do you know someone that has been infected with COVID-19?*	Yes/No
Proximity Symptomatic Person	*Are you close to someone with COVID-19 symptoms (fever, tiredness…)?*	Likert 5 Points, Never-Every day
Proximity Infected Person	*Are you close to individuals that are regularly in contact with infected populations?*	Likert 5 Points, Never-Every day
COVID-19 Symptoms	*Do you have any COVID-19 symptoms?*	Likert 5 Points, Never-Every day
COVID-19 Fear	*Are you scared of being infected by COVID-19?*	Range from 0–100 with 0 = Not Scared and 100 = Absolutely Scared
COVID-19 Information Frequency	*During lockdown, how frequently did you watch, read or listen to information about COVID-19?*	Likert 5 Points, Never-Several Times a Day
Child Screen Time	*How much time, on a daily average, does your child spend on TV, computers, mobile phones, or video games?*	Number of hours
Child Sleep Impact Family	*What is the degree of impact of your child's sleep quality on other family members' sleep?*	Range from 0–100 with 0= No impact and 100= Important Impact
Mother Number of Outings	*How often do you take time to go outside?*	Likert 5 Points, Never-Every day
Child Number of Outings	*How often is your child taken outside?*	Likert 5 Points, Never-Every day
Number of Times Child Sees Peers	*How frequently does your child see other children, apart from his/her siblings?*	Likert 5 Points, Never-Every day
Financial Impact	*Did the lockdown have a financial impact for your family?*	Range from 0–100 with 0 = No Impact and 100 = High Impact
Type of Housing [2]	*During the lockdown, you lived in: the city, the countryside, a house, an apartment; and, with a garden, with a balcony?*	Multiple responses possible

Note: [1] Recoded ordinally as −1 (positive), 0 (neutral), 1 (negative) impact based on the valence of the participant's free response (mixed responses=0)
[2] Coded ordinally 0–3 as Metropolitan Housing no private external area (PEA), Metropolitan with PEA, Countryside no PEA, Countryside with PEA

Table S2. Description and Preferred Threshold of SEM Fit Indices.

Indices	References	Description	Sensitive to Sample Size	Penalty for Complexity	Acceptable Threshold	Ideal Threshold
χ^2/df	(Jöreskog, 1969) [73]	A measure based on the covariance matrix deviation between the fitted model vs. the baseline model, divided by model degrees of freedom (*df*)	Yes	Yes	<5.0	-
Comparative Fit Index (CFI) [1,2]	(Bentler, 1990) [74]	An incremental measure based on a non-centrality statistic. Specifically, the ratio of the respective χ^2-*df* values of the fitted and baseline models.	No	Yes	>0.90	>0.95
Bollen's Incremental Fit Index (IFI) [2]	(Bollen, 1989 [76]; Marsh, Balla, & McDonald, 1988 [75])	The χ^2 difference between the fitted and baseline models, divided by the baseline model's χ^2-*df* result	Small *N*	Yes	>0.90	>0.95
Tucker Lewis Index (TLI) [1,2] Also called, Bentler Bonett Non-Normed Fit Index (NNFI)	(Tucker & Lewis, 1973) [77]	χ^2/df difference between the fitted and baseline models. Preferred over the NFI, a previously proposed index, which did not penalise for complexity.	No	Yes	>0.90	>0.95
Root Mean Squared Error of Approximation (RMSEA) [1,2]	(Steiger & Lind, 1980) [78]	The fitted model's χ^2-*df*, divided by its *df* times the sample size *N*.	Small *N*	Yes	<0.10	<0.08
RMSEA *p* Close Fit [1,2]	(Maccallum, Browne, & Sugawara, 1996) [79]	A one-sided test of the null hypothesis that the RMSEA equals .05. Hence, $p > 0.05$ is interpreted as a close fit of the model to the data.	Yes	Yes	>0.05	-
Goodness of Fit Index (GFI) [1,2]	(Jöreskog & Sörbom, 1981) [80]	Fit index based on the covariance matrix residuals. As known to be excessively affected by sample size, should be interpreted with caution.	Yes	No	>0.90	>0.95
Adjusted Goodness of Fit Index (AGFI) [1,2]	(Jöreskog & Sörbom, 1981) [80]	Fit index based on the nonredundant covariance matrix residuals. As known to be excessively affected by sample size, should be interpreted with caution.	Yes	Yes	>0.90	>0.95

Note: [1] (Schermelleh-engel, Moosbrugger, & Müller, 2003) [81]
[2] For a useful guide, see *http://davidakenny.net/cm/fit.htm*

Table S3. Descriptive Statistics for All Participants, French, and Swiss.

		Groups: French (F), Swiss (S)		
**Variables**	**All [1], N = 165**	**F [1], N = 81**	**S [1], N = 84**	**Significance [2] (F-S)**
Mother ISI Insomnia	10.05 (5.80)	11.30 (5.86)	8.86 (5.51)	**
Child DIMS Insomnia	18.44 (6.48)	19.81 (7.66)	17.11 (4.78)	**
Lockdown Impact	29%$^+$(34%$^-$)	20%$^+$ (42%$^-$)	38%$^+$ (27%$^-$)	*
Child Sex [3]	46%	36%	56%	*
Child Sees Peers	1.88 (1.11)	1.68 (0.96)	2.1 (1.21)	*
Child Sleep Impact Family	25.73 (32.10)	31.54 (35.36)	20.12 (27.67)	*
Mother Age	35.90 (4.29)	35.14 (3.81)	36.63 (4.62)	*
Child Number of Outings	4.02 (1.26)	3.70 (1.49)	4.32 (0.91)	*
Education Level	1.90 (1.50)	2.19 (1.25)	1.62 (1.66)	^
COVID-19 Fear	36.40 (29.64)	40.57 (32.15)	32.38 (26.59)	^
Relative Infected	64%	57%	70%	^
Mother STAI-B Anxiety	46.79 (7.29)	47.72 (7.35)	45.90 (7.17)	
Home Schooling	32%	38%	26%	
COVID-19 Information Seeking	2.02 (0.93)	1.90 (0.89)	2.12 (0.96)	
Child CGI-P Behaviour Scale	18.76 (5.94)	19.36 (5.89)	18.18 (5.96)	
COVID-19 Information Frequency	2.41(0.94)	2.30 (1.01)	2.5 (0.87)	
Mother Infection Risk	10%	14%	7%	
Working from Home	60%	56%	64%	
Mother Number of Outings	3.81 (1.18)	3.68 (1.27)	3.99 (1.07)	
Proximity Symptomatic Person	0.76 (1.29)	0.67 (1.20)	0.86 (1.36)	
Financial Impact	17.58 (24.94)	19.14 (25.02)	16.07 (24.91)	
COVID-19 Symptoms	0.69 (1.23)	0.63(1.18)	0.75 (1.28)	
Child Age	33.35 (15.17)	32.54 (14.62)	34.13 (15.73)	
Proximity Infected Person	0.97 (1.41)	1.05 (1.40)	0.89 (1.42)	
Type of Housing	0.42 (1.74)	0.42 (1.75)	0.43 (1.75)	
Num People During Lockdown	3.72 (0.79)	3.70 (0.73)	3.74 (0.85)	
Child Screen Time	0.83 (1.06)	0.82 (1.07)	0.83 (1.05)	

Note: [1] Mean (SD) or Frequency (%)
[2] Student t-test for continuous/interval variables, Wilcoxon rank sum test for ordinal variables, and χ2 frequency test for binary variables
[3] Binary variable coded 1 = Female, 0 = Male, Frequency (%) Female here provided
*Significance levels *** $p < 0.001$, ** $p < 0.01$, * $p < 0.05$, ^ $p < 0.10$*
The variables are ordered based on the most different between the countries versus the least different. The first line delineates the variables that are significantly different between the Swiss and French, the second for nonsignificant variables.
In the interest of brevity, Mean (SD) or Frequencies of each level of ordinal variables are not provided.

Table S4. Descriptive Statistics for the French Clusters.

Groups: Cluster 1 (C1), Cluster 2 (C2)			
**Variables**	**C1 [1], N = 42**	**C2 [1], N = 39**	**Significance [2] (C1-C2)**
Child DIMS Insomnia	−0.55 (0.81)	0.55 (0.84)	***
Child Sleep Impact Family	−0.50 (0.87)	0.50 (0.85)	***
Financial Impact	−0.39 (0.89)	0.33 (0.98)	***
COVID-19 Fear	−0.47 (0.98)	0.50 (0.75)	***
Child Number of Outings	0.47 (0.82)	−0.54 (0.93)	***
Child CGI-P Behaviour Scale	−0.36 (0.91)	0.34 (0.96)	**
Home Schooling	0.28 (1.02)	−0.42 (0.83)	**
Child Number of Times Sees Peers	0.27 (1.01)	−0.38 (0.87)	**
Mother ISI Insomnia	−0.30 (0.92)	0.25 (1.03)	*
Mother Infection Risk	−0.37 (0.42)	0.27 (1.21)	*
Mother STAI-B Anxiety	−0.28 (1.07)	0.20 (0.84)	*
Mother Education Level	0.29 (0.96)	−0.26 (0.98)	*
COVID-19 Information Seeking	0.22 (0.99)	−0.15 (0.98)	^
Lockdown Impact	26%$^{+}$ (33%$^{-}$)	13%$^{+}$ (49%$^{-}$)	^
Child Screen Time	−0.16 (0.99)	0.19 (1.01)	
COVID-19 Symptoms	−0.15 (0.90)	0.18 (1.10)	
Mother Tiredness	−0.15 (1.07)	0.16 (0.91)	
Number of People During Lockdown	0.13 (0.87)	−0.15 (1.03)	
Working from Home	0.21 (0.98)	−0.06 (1.01)	
Proximity Infected Person	−0.11 (0.97)	0.06 (1.03)	
Know Infected Person	−0.07 (1.01)	0.12 (0.99)	
Proximity Symptomatic Person	−0.18 (0.91)	0.08 (1.04)	
Child Age	−0.13 (1.06)	0.03 (1.00)	
Type of Housing	0.09 (1.00)	0.04 (1.01)	
COVID-19 Information Frequency	−0.02 (0.96)	−0.05 (1.04)	

Note: [1] Mean (SD) or Frequency %
[2] Student t-test for continuous/interval variables, Wilcoxon rank sum test for ordinal variables, and χ2 frequency test for binary variables
*Significance levels *** $p < 0.001$, ** $p < 0.01$, * $p < 0.05$, ^ $p < 0.10$*
The variables are ordered based on the most different between the clusters versus the least different. The first line delineates the variables that are significantly different between the clusters, the second for nonsignificant variables.
In the interest of brevity, Mean (SD) or Frequencies of each level of ordinal variables are not provided.

Table S5. Descriptive Statistics for the Swiss Clusters.

Groups: Cluster 1 (C1), Cluster 2 (C2)			
**Variables**	**C1 [1], N = 52**	**C2 [1], N = 32**	**Significance [2] (C1-C2)**
Lockdown Impact	54%$^+$ (15%$^-$)	12%$^+$ (47%$^-$)	***
Child Screen Time	−0.51 (0.87)	0.68 (0.73)	***
Child Age	−0.32 (0.99)	0.37 (0.85)	***
Mother Infection Risk	−0.28 (0.00)	0.45 (1.53)	***
Mother Education Level	0.50 (0.75)	−0.71 (0.93)	***
Working from Home	0.39 (0.77)	−0.74 (0.99)	***
Child Number of Outings	0.32 (0.83)	−0.48 (1.07)	***
Financial Impact	−0.15 (0.98)	0.34 (0.99)	*
Home Schooling	−0.25 (0.84)	0.27 (1.10)	*
COVID-19 Symptoms	0.18 (1.08)	−0.29 (0.81)	*
Number of People During Lockdown	−0.18 (0.97)	0.17 (0.88)	^
Child Number Times Sees Peers	0.23 (1.00)	−0.17 (0.95)	^
Mother ISI Insomnia	−0.23 (0.99)	0.14 (0.98)	^
Child Sleep Impact Family	−0.17 (1.01)	0.17 (0.95)	
Proximity Infected Person	0.14 (1.07)	−0.08 (0.97)	
COVID-19 Information Frequency	−0.09 (0.90)	0.07 (1.03)	
COVID-19 Fear	0.04 (1.02)	−0.16 (0.98)	
Child CGI-P Behaviour Scale	−0.11 (0.97)	0.07 (1.05)	
Know Infected Person	0.06 (0.98)	−0.10 (1.05)	
Mother Tiredness	−0.05 (1.06)	0.08 (0.90)	
Child DIMS Insomnia	−0.02 (0.86)	0.10 (1.01)	
Proximity Symptomatic Person	0.01 (1.01)	−0.05 (0.99)	
Mother STAI-B Anxiety	−0.10 (0.94)	−0.05 (1.06)	
COVID-19 Information Seeking	−0.05 (0.99)	0.01 (1.06)	
Type of Housing	0.00 (1.0)	0.07 (0.99)	

Note: [1] Mean (SD) or Frequency (%)
[2] Student t-test for continuous/interval variables, Wilcoxon rank sum test for ordinal variables, and $\chi 2$ frequency test for binary variables
*Significance levels *** $p < 0.001$, ** $p < 0.01$, * $p < 0.05$, ^ $p < 0.10$*
The variables are ordered based on the most different between the clusters versus the least different. The first line delineates the variables that are significantly different between the clusters, the second for nonsignificant variables.
In the interest of brevity, Mean (SD) or Frequencies of each level of ordinal variables are not provided.
